# Assessment of Anteroposterior Diameter and Interpedicular Distance in the Approximation of Cervical Spinal Canal Area

**DOI:** 10.7759/cureus.64244

**Published:** 2024-07-10

**Authors:** Zachary Brandt, Kai Nguyen, Paddington Mbumbgwa, Jacob Hauser, Rohan Kubba, Mark Oliinik, Andrew Fay, Asael Isaac, Jacob Razzouk, Gideon Harianja, Jun Ho Chung, Olumide Danisa, Wayne Cheng

**Affiliations:** 1 School of Medicine, Loma Linda University, Loma Linda, USA; 2 Department of Orthopaedic Surgery, Loma Linda University Medical Center, Loma Linda, USA; 3 Division of Orthopaedic Surgery, Jerry L. Pettis VA Medical Center, Loma Linda, USA

**Keywords:** central canal stenosis, artificial intelligence, shape approximations, approximation calculations, cervical spine

## Abstract

Rationale and objectives

This study seeks to generate a model based on two linear measurements, anteroposterior (AP) diameter and interpedicular (IPD) distance, to approximate the cervical central canal (CCC) area in a non-pathologic patient population by employing area calculations of shapes such as ellipse, rectangle, and triangle. Secondarily, this study aims to generate second-order approximations (SOAs), using the aforementioned shape approximations, to increase the utility of this linear measurement-based model.

Methods

The authors reviewed medical and radiographic records of patients aged 18-35 who received computed tomography (CT) imaging of the cervical spine to collect AP diameter, IPD distance, and area of the CCC from C2-3 to C6-7. Subsequently, shape approximations were calculated for each patient at all cervical spine levels. Lastly, SOAs were generated by combining optimal ratios of shape approximations to improve the statistical reliability of approximations.

Results

The ellipse shows the closest approximation to manual measurements of the individual shape approximations. Percent error analysis demonstrated the superiority of the ellipse, followed by the rectangle, and lastly the triangular approximation. The highest correlation of approximations was observed at C6-7. All individual shape approximations demonstrated statistical differences from manual measurements. SOAs combining ellipse and rectangle measurements demonstrated superior accuracy and were not statistically different from manual measurements.

Conclusion

Individual shape approximations based on AP diameter and IPD distance show some value as a model for the assessment of the CCC area. SOAs demonstrated greater utility than individual shape approximations and show promise as a linear measurement-based tool to assess the CCC area.

## Introduction

The prevalence of cervical spine pathology is increasing with the aging global population, resulting in significant morbidity that affects communities worldwide [1,2]. Conditions involving the cervical spine are primarily attributed to age-related degeneration and can entail considerable morbidity [3]. In particular, central canal stenosis of the cervical spine is associated with a variety of debilitating neurologic symptoms that warrant consideration for surgical intervention [4-6]. Although there are many ways to assess for central canal stenosis, imaging modalities such as X-ray, magnetic resonance imaging (MRI), and computed tomography (CT) are the most common. These modalities assess the area of the spinal canal and investigate the severity of central canal stenosis.

Medical science is progressing rapidly, facilitating swifter patient diagnosis and accelerated treatment. Among the various strides made in this field, the incorporation of artificial intelligence (AI) into radiologic interpretations stands out. The application of AI has demonstrated its efficacy in expediting the reading and interpretation process in numerous medical scenarios [5,7]. One such advancement in the medical field would be the implementation of AI in the assessment of the cervical central canal (CCC) area.

AI evaluation of the central canal area can occur through various approaches. Due to complexities associated with the direct assessment of the central canal area, Brandt et al. proposed a model built on approximations that AI could use [8-11]. This model involves employing two linear measurements, the anteroposterior (AP) diameter and interpedicular (IPD) distance, to calculate the area using various shapes such as an ellipse, rectangle, and triangle as approximations for the shape of the spinal central canal. The rationale behind this approach is twofold. First, AI has demonstrated remarkable accuracy in linear anatomic measurements achieving rates of up to 98.95% [10]. Second, these shape calculations were shown to be highly correlated with manual measurements in the lumbar central canal [11]. This model was constructed with the linear accuracy AI anatomic measurements and previously demonstrated correlation values in mind.

An additional aspect of this research involves evaluating the effectiveness of combining shape approximations also known as second-order approximation (SOA). These SOAs intend to achieve greater accuracy than individual shape approximations. Previous research using individual shape approximations demonstrated that they statistically differed from manual measurements at all lumbar spine levels [11]. Using an SOA, the authors seek to develop approximations based on AP diameter and IPD distance that do not statistically differ from manual measurements.

## Materials and methods

Following institutional review board approval from Loma Linda University Health Human Research and Compliance (approval number: 5240112), the authors reviewed medical and radiographic records of patients between 18 and 35 years of age who received CT imaging of the cervical spine between January 2015 and March 2023. Potential participants were systematically assessed based on the chronological order of their completed imaging. Exclusion criteria encompassed individuals with a history of pathologic conditions. This included anatomic deformities such as disc degeneration, scoliosis, and spondylolisthesis. Additionally, patients with a history of traumatic spinal injury, spinal abscess, malignancy in the spine, pain of neurologic origin in the back and legs, numbness, spinal instrumentation, or prior spinal surgery were also excluded from the study. The reason for such exclusion criteria revolves around developing an appropriate non-pathologic population. Having a relevant "normal" population allows for establishing a baseline functionality of the constructed approximations. Anatomic studies by Razzouk et al. and Nguyen et al. demonstrate the importance of establishing a baseline in a similar patient population [12,13].

**Figure 1:**
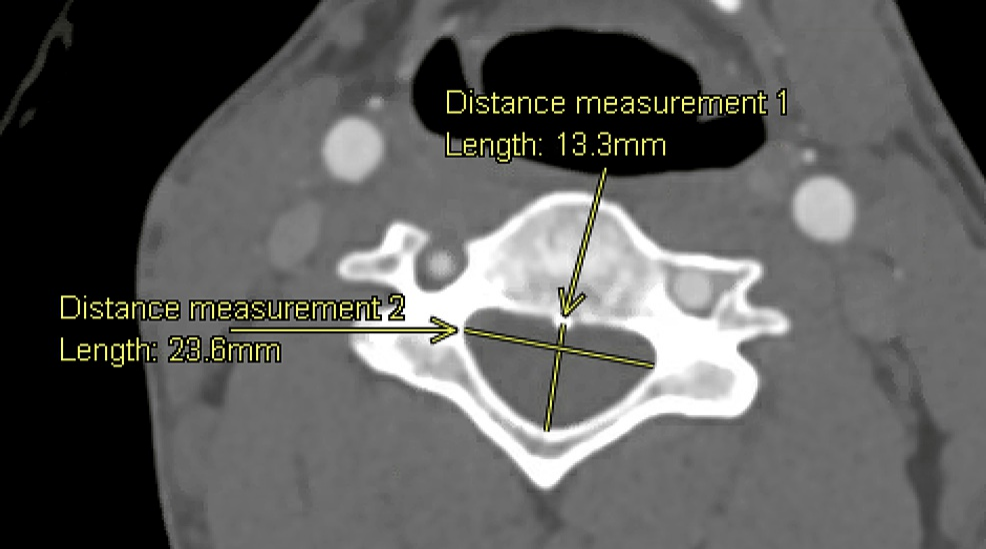
AGFA Enterprise Imaging measurements of AP diameter (Distance measurement 1) and IPD distance (Distance measurement 2) AP: anteroposterior; IPD: interpedicular

**Figure 2:**
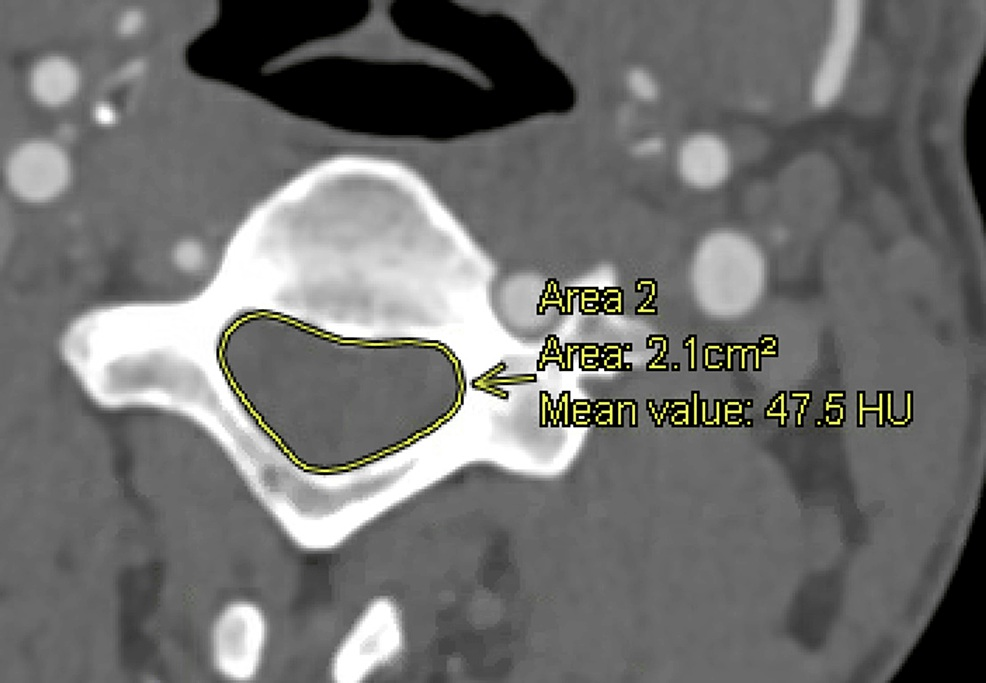
AGFA Enterprise Imaging measurements of the spinal canal area

AP diameter, IPD distance, and spinal canal area of the cervical spine, C2-7, were measured in patients using AGFA HealthCare's Enterprise Imaging 8.3.x platform. Board-certified neuroradiologists trained the authors to conduct the previously described measurements. Figure 1 illustrates measurements of the AP diameter and IPD distance, while Figure 2 depicts the measurement of the spinal canal area. IPD distance was defined as the maximum distance between the medial aspect of the pedicles at a specified vertebral level. The canal area was determined using the AGFA Enterprise Imaging markup freeform tool. Following this, an estimated area of the CCC, assuming an elliptical shape, was computed using Equation 1: area=(π)×(1/2)(IPD)×(1/2)(AP diameter). Triangular and rectangular approximations were performed using Equations 2 and 3, respectively: area=(1/2)(IPD)×(AP diameter) and area=(IPD)×(AP diameter). SOA equations were calculated using a line of best fit between measured values and a combination of elliptical and rectangular approximations. Equations at each cervical spinal level were optimized until a paired sample t-test displayed no statistical significance between measured and approximated. SOA equations are displayed in Equations 4-8: area=(1)(ellipse approximation)×(0.07091)(rectangle approximation)​​​​​​​, area=(1)(ellipse approximation)×(0.046525)(rectangle approximation)​​​​​​​, area=(1)(ellipse approximation)×(0.041866)(rectangle approximation), area=(1)(ellipse approximation)×(0.0329399)(rectangle approximation), and area=(1)(ellipse approximation)×(0.0210019)(rectangle approximation)​​​​​​​.

Statistical analyses

Statistical analyses were conducted similarly to Brandt et al. as this proved effective in the evaluation of shape approximations of the lumbar central canal [11]. IBM SPSS Statistics for Windows, Version 28.0 (Released 2021; IBM Corp., Armonk, New York, United States) was the primary tool for statistical analyses. Alpha was defined as p<0.05. To assess the normality of data, Kolmogorov-Smirnov tests and Q-Q plots were conducted. Homoscedasticity was assessed by residual regression plots and Levene's homogeneity of variance test. Pearson correlation tests were used to assess associations between measurements and resulting approximations. Weak, moderate, and strong coefficients were defined as 0-0.4, 0.4-0.7, and 0.7-1, respectively. There were several descriptive statistics collected including averages, standard deviations (SD), ranges, mean differences (MD), and 95% confidence intervals (CI). To assess if statistically significant differences exist between measured and approximated values, a paired sample t-test was performed. Lastly, to further evaluate the accuracy of approximations, mean differences and percent error of approximations from manual measurements were calculated. 

## Results

A total of 803 patients were included in the study with a mean age of 26.06±5.88 years. The average height, weight, and BMI were 1.69±0.11 meters (m), 82.27±24.47 kilograms (kg), and 27.95±8.00 kg/m^2^, respectively. There were a total of 369 females and 434 males. The authors' manual measurements revealed that the average area of the spinal canal ranged from 257.49 mm² at C3-4 to 280.72 mm² at C2-3. Detailed average areas for each cervical vertebra are provided in Table 1. Among the shape approximations, the ellipse exhibited the closest approximation to the spinal canal area, with mean differences ranging from -7.51 mm² at C6-7 to -23.18 mm² at C2-3. The rectangle calculation showed the second most accurate approximation, with mean differences ranging from 47.19 mm² at C2-3 to 64.58 mm² at C6-7. Conversely, the triangular approximation displayed the highest mean differences, ranging from -116.77 mm² at C2-3 to -102.84 mm² at C3-4. Importantly, all measured differences were statistically significant (p<0.001), as depicted in Table 2.

**Table 1:** Average measurement values

Level	Manual measurement	Ellipse	Triangle	Rectangle
C2-3	280.72±71.50	257.54±65.51	163.95±41.70	327.91±83.41
C3-4	257.49±46.32	242.93±62.86	154.65±40.02	309.30±80.03
C4-5	265.99±45.51	252.55±65.58	160.78±41.75	321.56±83.50
C5-6	273.83±48.64	262.49±72.50	167.10±46.15	334.21±92.31
C6-7	271.38±57.14	263.86±78.58	167.98±50.03	335.96±100.05

**Table 2:** Mean differences compared to the manual measurement of the spinal canal area P-values were calculated by paired sample t-test

Level	Shape	Mean difference (mm²)	p	Correlation
C2-3	Ellipse	-23.18±61.41	<0.001	0.601
	Triangle	-116.77±57.14	<0.001	0.601
	Rectangle	47.19±69.98	<0.001	0.601
C3-4	Ellipse	-14.57±52.44	<0.001	0.575
	Triangle	-102.84±40.20	0.001	0.575
	Rectangle	51.81±65.49	<0.001	0.575
C4-5	Ellipse	-13.44±56.06	<0.001	0.541
	Triangle	-105.21±41.93	<0.001	0.541
	Rectangle	55.57±70.23	<0.001	0.541
C5-6	Ellipse	-11.34±56.77	<0.001	0.624
	Triangle	-106.73±41.17	<0.001	0.624
	Rectangle	60.38±72.70	<0.001	0.624
C6-7	Ellipse	-7.51±56.17	<0.001	0.700
	Triangle	-103.40±42.03	<0.001	0.700
	Rectangle	64.58±72.62	<0.001	0.700

The ellipse approximation exhibited the lowest percent error, ranging from -2.77% at C6-7 to -8.26% at C2-3. Following suit, the rectangular approximation emerged as the second most accurate, with percent errors ranging from 18.81% at C2-3 to 23.80% at C6-7. Conversely, the triangular approximation displayed percentage errors ranging from -38.10% at C6-7 to -41.60% at C2-3. Complete percent error values are provided in Table 3.

**Table 3:** Approximation percent error calculations compared to manual measurements

Level	Ellipse	Triangle	Rectangle
C2-3	-8.26%	-41.60%	16.81%
C3-4	-5.65%	-39.94%	20.12%
C4-5	-5.05%	-39.55%	20.89%
C5-6	-4.14%	-38.98%	22.05%
C6-7	-2.77%	-38.10%	23.80%

The approximations ranged from moderately to highly correlated with the authors' manual measurements. The most highly correlated approximations were located at the C6-7 vertebrae with a value of 0.700. This was closely followed by approximations at C5-6 with a value of 0.624. The lowest correlated approximations were at the C4-5 vertebrae level and had a value of 0.541. Table 2 contains all correlation values.

SOAs were computed for each level given the variability in shape approximation accuracy across cervical vertebrae levels. Notably, at C4-5, the SOA proved to be the most accurate, with a mean difference of 0.02 mm², whereas at C5-6, it demonstrated the least accuracy, with a mean difference of -0.33 mm². Importantly, all p-values exceeded 0.05. All SOA values are in Table 4.

**Table 4:** Manual measurements vs. second-order approximation (mm²) P-values were calculated by paired sample t-test

Level	Second order	Mean difference	Percent error	Correlation	p
C2-3	280.79±71.42	0.07±63.78	15.06%	0.601	0.98
C3-4	257.35±66.59	-0.21±54.95	17.89%	0.575	0.91
C4-5	266.06±69.11	0.02±58.63	18.63%	0.541	0.99
C5-6	273.50±75.53	-0.33±59.02	18.31%	0.624	0.87
C6-7	271.92±79.12	-0.05±56.33	17.56%	0.700	0.98

## Discussion

This study pursued two primary objectives. Initially, it aimed to evaluate the efficacy of elliptical, triangular, and rectangular approximations in the assessment of CCC area. Subsequently, it aimed to develop and validate an SOA utilizing two or more of these shape approximations that would not statistically differ from manual measurements. The precision observed in prior studies concerning AI anatomical approximations influenced the decision to employ a two-linear measurement approximation [14]. These measurements facilitated the computation of various shapes. The selection of shapes for approximation was based on two factors: firstly, on existing literature describing the spinal canal's shape, and secondly, on shapes capable of consistently either overestimating or underestimating area to enhance the practicality of a SOA [15,16]. Multiple factors influence the effectiveness of the authors' approximation of the spinal canal. Key considerations for evaluating this approximation include the mean difference of each approximation, percentage error, correlation values, and the presence of any statistically significant difference between the approximation and manual measurements.

When assessing the three shape approximations, ellipse, triangle, and rectangle, consistent mean differences were observed compared to manual measurements at each level, based on shape. The ellipse approximation demonstrated the highest accuracy across all levels, consistently underestimating the canal area. As measurements progressed caudally, the mean difference increased, converging toward zero, indicating a more elliptical shape of the central canal. This finding aligns with previous studies exploring normative measurements of the CCC [17,18]. Conversely, the rectangle approximation, the second most accurate, consistently overestimated the spinal canal area. Its highest accuracy was at the cephalic end of the cervical spine, with accuracy decreasing as measurements moved caudally. Finally, the triangle approximation emerged as the least accurate among all the approximations. It demonstrated its highest accuracy at the C3-4 level but its lowest accuracy at C2-3, without showing a consistent pattern from cranial to caudal levels. The inconsistency aligns with the cervical spine's lack of a distinct triangular pattern, which is observed in the lumbar spine and particularly evident as it descends caudally [16]. All mean difference values can be observed in Table 1.

The percentage error analysis reveals the elliptical approximation's strong suitability for cervical spine assessments. Throughout the analysis, the percentage error for the ellipse approximation never exceeds 9% and improves in effectiveness as it descends caudally. With an error rate as minimal as -2.77%, the ellipse approximation demonstrates a level of accuracy comparable to manual measurements. It's worth noting that factors such as partial volume effect, beam hardening, machine calibration, and spatial resolution can introduce inherent imaging errors of up to ±6% further illustrating the accuracy of the ellipse approximation [19]. On the other hand, despite being the second most accurate, the rectangular approximation presents less promising outcomes. Its percentage error ranges between 16.81% and 23.80%, limiting its clinical applicability when used independently. However, the rectangular approximation holds significant value in an SOA framework due to its tendency to overestimate. Lastly, the triangular approximation exhibits the highest percentage error, consistently underestimating the central canal's area by significant margins. With error values exceeding -40%, the triangular approximation's utility is compromised both as a standalone approximation and in an SOA context. All percentage error values can be observed in Table 3.

When assessing the correlation values for each shape approximation, it is clear that correlation does not vary based on approximation but rather upon cervical spinal level. This is because all approximations are based on the AP diameter and IPD distance collected at those levels. The level of correlation is moderate at the vast majority of levels including C2-3 to C5-6, with a high level of correlation at C6-7. All values for the correlation of shape approximations can be observed in Table 2, while SOA correlations are in Table 4.

Lastly, evaluation of each shape approximation via statistical analysis is critical to assess the utility of the approximation on a patient-by-patient basis. With a sample size as large as is present in this study, it becomes mathematically difficult to create approximations that will not demonstrate statistical differences [20]. As expected, each shape approximation at each cervical spine level demonstrates a statistically significant difference between the manual measurements (p<0.001). All values can be observed in Table 2. Due to this difference between the manual measurements and each approximation, the authors were commissioned to create an SOA that eliminated the statistical difference between approximation and manual measurements.

SOAs are a common mathematical tool that is used in broad areas of life such as economics, finance, physical chemistry, and engineering [21-23]. Although the terminology for SOAs is inconsistent depending on the field, the basic principle revolves around using two variables or a polynomial to more accurately calculate the desired value. This study used the ellipse and rectangle approximation as the two variables to more accurately predict the manual measurements. SOA accuracy was greatest when the ellipse approximation's full value was combined with a proportion of the rectangular approximation. This use of the full proportion of the ellipse approximation was possible due to the consistent underestimation of the true area of the CCC by the elliptical approximation. The rectangular approximation was used to bridge this gap as it consistently overestimated the value of the CCC. A best-fit calculation based on the cervical spine level was performed and produced Equations 4-8. These SOAs were subsequently evaluated in the same manner as the previous shape approximations.

The SOA consistently demonstrated superior accuracy across most metrics and cervical spine levels compared to primary shape approximations. The mean difference between the SOA and manual measurements remained consistently below 0.5 mm², ranging from -0.33 mm² to 0.02 mm². Although the percent error ranged from 15.06% to 18.63%, the SOA outperformed both rectangle and triangle approximations, albeit falling short of the ellipse approximation. Since the SOA was also based on the AP diameter and IPD distance, correlation values remained consistent with other approximations, with the highest correlation observed at C6-7 (0.700). Notably, the most significant achievement of the SOA was evident in statistical analysis, as it exhibited no difference from manual measurements across all cervical spine levels, with p-values ranging from 0.87 to 0.99.

Developing an approximation method that aligns statistically with gold-standard manual measurements opens up numerous future applications. When combined with AI capable of conducting linear measurements such as AP diameter and IPD distance, these approximations can accurately calculate a patient's CCC area. Several studies have already utilized AI for taking linear measurements akin to those discussed here, encompassing various cervical spine parameters including sagittal vertical axis (SVA), C0-C2 distance, Redlund-Johnell distance (RJD), cranial tilting (CT), craniocervical angle (CCA), C7 slope, and T1 slope [24,25]. The effective performance of these measurements by AI underscores the potential of the approximation equations proposed by the authors. If a similar model driven by AI could be integrated into imaging modalities, patients at risk of central canal stenosis could be promptly identified, enabling physicians to maintain a high level of suspicion for this condition.

Limitations

This study has several limitations that need consideration. Firstly, the findings may only apply to patients who fall within the inclusion criteria employed in this research. Patients with spinal stenosis often involve spinal canal deformities, which are not accounted for in the approximation equations used in this study. However, this was intentional this project sought to test the functionality of approximations in a baseline population. Additionally, measurements were obtained by multiple observers, potentially introducing additional errors in the approximations. Lastly, the SOA was devised and tested to prove intrinsic validity. The approximation needs to be applied to an outside group of patients to ensure statistical validity remains true.

## Conclusions

Shape approximations based on AP diameter and IPD distance show promise as an effective model for AI-driven assessment of the CCC area. The authors' development of an SOA that aligns statistically with manual measurements presents numerous avenues for future exploration and implementation. Further exploration of these approximations across external datasets and in patients diagnosed with central canal stenosis could broaden the clinical relevance of this research.
